# A *nox2/cybb* zebrafish mutant with defective myeloid cell reactive oxygen species production displays normal initial neutrophil recruitment to sterile tail injuries

**DOI:** 10.1093/g3journal/jkae079

**Published:** 2024-05-02

**Authors:** Abdulsalam I Isiaku, Zuobing Zhang, Vahid Pazhakh, Graham J Lieschke

**Affiliations:** Australian Regenerative Medicine Institute, Monash University, Clayton, VIC 3800, Australia; Australian Regenerative Medicine Institute, Monash University, Clayton, VIC 3800, Australia; Australian Regenerative Medicine Institute, Monash University, Clayton, VIC 3800, Australia; Australian Regenerative Medicine Institute, Monash University, Clayton, VIC 3800, Australia; Department of Clinical Haematology, Peter MacCallum Cancer Center and The Royal Melbourne Hospital, Parkville, VIC 3050, Australia

**Keywords:** *nox2*, *cybb*, ROS, acute inflammation, neutrophils, wound, chemotaxis, zebrafish, chronic granulomatous disease

## Abstract

Reactive oxygen species are important effectors and modifiers of the acute inflammatory response, recruiting phagocytes including neutrophils to sites of tissue injury. In turn, phagocytes such as neutrophils are both consumers and producers of reactive oxygen species. Phagocytes including neutrophils generate reactive oxygen species in an oxidative burst through the activity of a multimeric phagocytic nicotinamide adenine dinucleotide phosphate oxidase complex. Mutations in the *NOX2/CYBB* (previously gp91^phox^) nicotinamide adenine dinucleotide phosphate oxidase subunit are the commonest cause of chronic granulomatous disease, a disease characterized by infection susceptibility and an inflammatory phenotype. To model chronic granulomatous disease, we made a *nox2/cybb* zebrafish (*Danio rerio*) mutant and demonstrated it to have severely impaired myeloid cell reactive oxygen species production. Reduced early survival of *nox2* mutant embryos indicated an essential requirement for *nox2* during early development. In *nox2/cybb* zebrafish mutants, the dynamics of initial neutrophil recruitment to both mild and severe surgical tailfin wounds was normal, suggesting that excessive neutrophil recruitment at the initiation of inflammation is not the primary cause of the “sterile” inflammatory phenotype of chronic granulomatous disease patients. This *nox2* zebrafish mutant adds to existing in vivo models for studying reactive oxygen species function in myeloid cells including neutrophils in development and disease.

## Introduction

Neutrophil granulocytes are major initial responders to infection and inflammation. Many neutrophil functions are regulated by intracellularly generated reactive oxygen species (ROS). These include chemotaxis, phagocytosis, and neutrophil extracellular trap release. ROS production by neutrophils is generated by an active process involving the phagocyte nicotinamide adenine dinucleotide phosphate (NADPH) oxidase (PHOX) enzyme. PHOX is a multisubunit enzyme with cytosolic (NCF1/p47^phox^, NCF2/p67^phox^, NCF4/p40^phox^) and transmembrane (CYBA/p22^phox^, NOX2/CYBB/gp91^phox^) subunit proteins (Panday *et al*. 2015; Quinn 2013; Thomas *et al*. 2017). CYBC1/EROS chaperones gp91^phox^ and p22^phox^ heterodimerization in the endoplasmic reticulum but is not part of the NADPH oxidase complex itself (Randzavola *et al*. 2022).

The importance of PHOX-derived ROS is highlighted by chronic granulomatous disease (CGD), a primary immune deficiency characterized by genetic mutation in one of the PHOX subunit proteins, severe recurrent infections, and/or excessive inflammation (Dinauer 2019). NOX2/CYBB is the most highly expressed PHOX subunit protein in neutrophils, and *NOX2/CYBB* mutations account for ∼65% of human CGD (Dinauer 2019). Although the vulnerability of CGD patients to infections is directly linked to a reduced level of neutrophil NOX2-derived ROS production, the exact mechanisms behind inflammatory manifestations of CGD are less clear (Marciano *et al*. 2018; Roos 2016).

Zebrafish models have made important contributions to understanding the roles of phagocyte-derived ROS. Several studies have used zebrafish morphants targeting *nox2/cybb* or other PHOX subunits, showing increased susceptibility to fungal and bacterial infections attributed to ROS deficiency (Brothers *et al*. 2011; Mesureur *et al*. 2017; Yang *et al*. 2012). This is consistent with the established view that ROS-dependent neutrophil antimicrobial effects are central to host defense against pathogens and demonstrates the conserved role of ROS in cell-autonomous innate immunity (Randow *et al*. 2013). Two zebrafish *nox2/cybb* mutant alleles have been previously described, but their effects on neutrophil ROS production and other neutrophil cellular functions have not been characterized (Terzi *et al*. 2021; Weaver *et al*. 2018).

Immediately after tissue injury, ROS levels represent a complex interplay between ROS generated by other tissues and ROS consumption and production by neutrophils. Tissue injury results in a burst of DUOX-dependent - hydrogen peroxide (H_2_O_2_) production by epithelial cells which acts as a neutrophil chemoattractant (Niethammer *et al*. 2009), and which is sensed in neutrophils by oxidation of the cysteine C466 residue in Lyn, an Src family kinase (Niethammer *et al*. 2009; Yoo *et al*. 2011). Arriving neutrophils down-regulate this H_2_O_2_ by a myeloperoxidase-dependent mechanism (Pase *et al*. 2012). However, activation of arriving neutrophils stimulates their oxidative burst, adding neutrophil-generated ROS to the inflammatory response. Understanding this dynamic interplay of ROS production, consumption, and decay requires tools that selectively modulate the various ROS contributions of different cells and enzymes.

We have generated and characterized a new *nox2/cybb* zebrafish mutant with myeloid cells displaying profoundly impaired ROS production. We also investigated the effect of their *nox2/cybb*-dependent ROS deficiency on initial neutrophil recruitment to acute surgical wounds but found that loss of *nox2*-derived ROS did not alter the numbers of neutrophils initially recruited to minor or severe acute wound injuries. This mutant is a new model of CGD and provides a tool for studying Nox2-dependent myeloid cell dysfunction characterizing CGD.

## Materials and methods

### Animal ethics and zebrafish alleles

Zebrafish strain used were wild type (WT) Tübingen (TU) (Max-Planck-Institut für Entwicklungsbiologie, Tübingen, Germany), *Tg(mpx:EGFP)^i114^* (Renshaw *et al*. 2006), the compound transgenic reporter line *Tg(mpx:Kal4TA4)^gl28^* (Okuda *et al*. 2015), *Tg(UAS-E1b:Eco:NfsB-mCherry)^c26^*^4^ (Davison *et al*. 2007), and *nox2/cybb^gl43^* (hereafter called *nox2^−/−^*) carried on both reporter backgrounds (this report). Zebrafish experiments were conducted under protocols approved by Monash University Animal Ethics Committees (MARP-2015-094/14375 and 17270) and in accordance with Australian Code of Practice for the Care and Use of Animals for Scientific Purposes (NHMRC 2021).

### Gene expression analysis

The publicly available EMBL-EBI Elixir node Single Cell Expression Atlas data and tools were employed using the following data sets (www.ebi.ac.uk/gxa/sc/home, accessed analysis 2024 January 12): “single-cell RNA-seq analysis of kidney marrow from 6 zebrafish transgenic lines that label specific blood cell types” *n* = 245 (Tang *et al*. 2017), “single-cell RNA-seq data of zebrafish blood cells data” *n* = 1,354 cells (Athanasiadis *et al*. 2017), and “single-cell RNA sequencing of the cut and uncut caudal fin of zebrafish larvae” *n* = 2,860 cells (data provided without published reference).

### CRISPR-Cas9 mutagenesis

Targeted mutation of *nox2* (Ensembl ENSDARG00000056; ZFIN: ZDB-GENE-040426-1380 (Bradford *et al*. 2022)) was achieved by microinjecting dual guide RNAs (gRNAs) complexed with Cas9 into 1-cell stage Tuebingen (TU) zebrafish embryos (Jao *et al*. 2013). The gRNAs targeted sequences adjacent to PAM sites in exons 3 and 4 of the *nox2* gene (5′-GCCCACGAGAGAGCATGCTG-3′ and 5′-CAAGCTGTCGAGCTGCAGTG-3′, respectively).

### PCR and RT-PCR

Genomic DNA was extracted using HotShot method (Meeker *et al*. 2007). PCR amplification of fragments spanning the gRNA target sites used the following primers: gRNA1 site, 5′-GGTTGTAAATGTGATGCCGTAA-3′ and 5′-AATTTCGGATACAGCCCAAGTA-3′, and gRNA2 site, 5′-TGCAATCATAATGAAAAGGGA-3′ and 5′-GCTGCAATTCTTAAATATCCGC-3′. PCRs used Phusion High-Fidelity DNA Polymerase (ThermoFisher Scientific) and a T100 thermal cycler (Bio-Rad).

RNA was extracted with TRIzol reagent. cDNA was synthesized using SuperScript VILO cDNA Synthesis kit (ThermoFisher Scientific). RT-PCR to detect *nox2* mis-splicing used the following primers: *nox2* (positioned in introns 2 and 3), 5′-GTATGGCTCGGGATCAATGTGT-3′ and 5′-GATCCTCGGAGAAACGAGAGC-3′, and *ppial*, 5′-ACACTGAAACACGGAGGCAAAG-3′ and 5′-CATCCACAACCTTCCCGAACAC-3′ (van der Vaart *et al*. 2013; van Soest *et al*. 2011).

### Sanger sequencing

The PCR products representing genomic and cDNA sequences were purified using PCR purification kit (Promega) and subsequently sequenced using Sanger sequencing at Micromon Genomics, Monash University.

### Quantification of ROS

ROS production in whole embryos was measured as previously described (Goody *et al*. 2013). Briefly, whole embryos of known genotype were divided into phorbol myristate acetate (PMA) and non–PMA-stimulated groups in a dark 96-well plate. The redox dye 2′,7′-dichlorodihydrofluorescein diacetate (H2CFDA) was added. Fluorescence readings were taken at room temperature after 30–60 min, every 12 min for 180 min using a spectrophotometer (Infinite M200 Pro, Tecan).

A ROS assay using dihydrorhodamine 123 (DHR123) was employed, as used in CGD diagnosis (Emmendorffer *et al*. 1994; Vowells *et al*. 1996). Phorbol 12-myristate 13-diacetate (PMA) was used to stimulate ROS production in FACS-gated myeloid cells obtained from WT and *nox2*^−/−^ zebrafish whole kidney marrow (WKM) prepared from nontransgenic backgrounds devoid of reporter genes. Unstimulated WKM myeloid cells served as control. The stimulation index was calculated by expressing the paired PMA stimulated:unstimulated fluorescence intensity ratio as a percentage. For FACS purification, single-cell preparations were prepared from dissected whole kidneys by mechanical disruption and strained through a 35 µm filter into FACS tubes. Dead cells were excluded by DAPI staining. The myeloid cell gate was determined by forward and side scatter flow analysis using BD LSRFortessa ×20 (Traver *et al*. 2003). Samples are WKM myeloid cells from single animals.

### Viability assay

Zebrafish embryos from 3 adult zebrafish crosses; *WT × WT*, *WT × nox2^−/−^* (mixed maternal genotypes), and *nox2^−/−^×nox2^−/−^* were observed at 24 h or daily for up to 5 days postfertilization (dpf). The numbers of live and dead embryos were counted manually.

### Wound assay

Two- to 3-day postfertilization embryos from *nox2^+/−^* incrosses were anesthetized using 160 mg/mL tricaine methanesulfonate (Sigma-Aldrich). Using a fine scalpel blade, 2 types of wound were created of different severity, excising either the tip of the tail fin alone or the tip of the tail fin along with the distal tip of the notochord. Images of individual embryos were taken using MVX10 microscope fitted with Olympus DP72 camera and cellSens software (version 1.11). The number of neutrophils at the wound site (caudal vein loop to transection point) was manually counted from images of embryos as previously described (Isiaku *et al*. 2021; Miskolci *et al*. 2019). Neutrophil numbers were scored blinded, prior to genotyping of embryos.

### Statistical analysis

GraphPad Prism version 8.3.1 was used for statistical analysis. A 2-way ANOVA with Tukey's multiple comparison or unpaired 2-tailed *t*-test was used to test the difference in ROS production. Mann–Whitney was used in testing differences in ROS stimulation index. Log-rank (Mantel–Cox) and Fisher's exact tests were employed to compare survival rates. A 2-way ANOVA with Geisser–Greenhouse correction and Tukey's multiple comparisons tests was used to assess the relationship of time and number of neutrophils between the 3 groups of different genotype-matched siblings. Equality of variance and sphericity were not assumed. *P* values < 0.05 were considered significant.

## Results

### Expression of zebrafish *nox2/cybb*

Zebrafish *nox2/cybb* (hereafter *nox2*) expression was evaluated using publicly available single-cell RNA-seq data sets with reference to expression of the neutrophil marker genes *mpx* and *lyz* and macrophage marker genes *mpeg1.1* and *mfap4* (Supplementary Fig. 1). Zebrafish *nox2* was expressed primarily in both phagocyte types, as expected of a phagocyte NAPDH oxidase (Supplementary Fig. 1a). It had a narrower cellular spectrum of expression than that of its transmembrane partner *cyba*/p22^phox^ (Supplementary Fig. 1b), consistent with the known functional partnership of Cyba with other Nox proteins (Quinn 2013). The cytosolic subunit *ncf1/p47^phox^* of phagocytic NAPDH oxidase displayed a similar pattern of expression. Within the heterogeneous population of cells in the zebrafish tail fin region (Supplementary Fig. 1b), *nox2* expression segregated to a myeloid cluster containing *mpx*-, *ly-z*-, *mpeg1.1*-, and *mfap4*-expressing cells, whereas *cyba* displayed expression not restricted to this myeloid cluster, and *nox1* expression localized to an alternative cluster characterized by expression of marker genes potentially indicating an epithelial-related identity (e.g. *cyt1*).

### A zebrafish *nox2* mis-splicing mutant

**Fig. 1.**
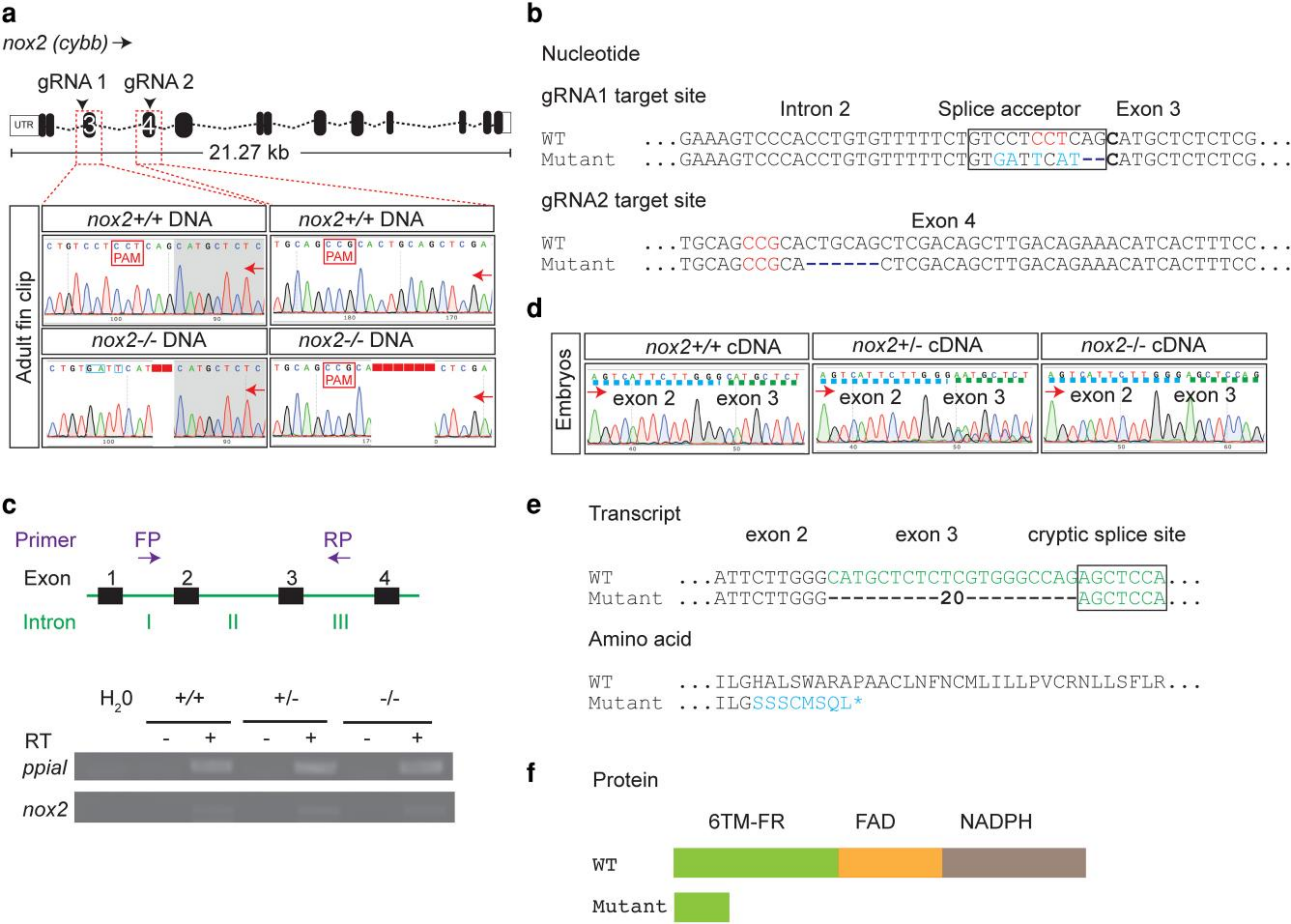
Genetic and molecular characterization of adult *nox2* zebrafish. a) Sanger sequencing chromatogram of genomic DNA at gRNA 1 (intron 2/exon 3) and gRNA 2 (exon 4) target sites in adult WT (upper panel) and *nox2* (lower panel) homozygous zebrafish. DNA sequence disruptions are around the protospacer adjacent motif (PAM)—NGG. b) Interpretation of nucleotide sequences in panel a, at the gRNA 1 and gRNA 2 target sites. c) Schematic of PCR forward primer (FP) and reverse primer (RP) binding sites for amplifying cDNA transcripts (above). Gel image showing RT-PCR products from *nox2* WT, heterozygous, and homozygous zebrafish embryos (below). d) Sanger chromatogram showing cDNA sequence traces of *nox2* WT, heterozygous, and homozygous zebrafish embryos from RT-PCR in panel c. Exons separated by colored broken lines. e) Interpretation of nucleotide sequence and predicted amino acid sequence of WT and *nox2* homozygous in panel d. f) Schematic of predicted protein domains in WT and *nox2* zebrafish. *nox2*, NADPH oxidase 2; gRNA, guide RNA; PAM, protospacer adjacent motif; 6TM-FR, heme containing 6 transmembrane ferritin reductase domain; FAD, flavine adenine dinucleotide domain; NADPH, nicotinamide adenine dinucleotide phosphate domain; +/+, WT; +/−, heterozygous; −/−, homozygous; green font, exon 3 sequence; blue font, sequence mismatch; asterisk (*), premature stop; purple arrows, direction of sequencing; FP, forward primer; RP, reverse primer; RT, reverse transcriptase.

To generate a zebrafish *nox2* mutant, 2 multiplexed gRNAs were delivered targeting PAM sites in intron 2 adjacent to the exon 3 splice acceptor and exon 4 of *nox2* (Fig. 1a). The gRNAs were designed intending to induce deletion of intervening sequences between the 2 PAM targets, but no stable mutants with large deletions were recovered. A majority of F1 animals recovered carried mutations at either 1 or both PAM targets.

A single mutant allele was selected for further studies. At the gRNA1 target site, there was a 2 base pair (bp) nucleotide deletion and 5 bp nucleotide substitution (Fig. 1, a and b). At the gRNA2 target site, there was a 6 bp deletion (Fig. 1, a and b). While the triplet deletion at the gRNA2 PAM did not alter the reading frame and was therefore of uncertain functional significance, the changes at the gRNA1 target site altered the exon 3 splice acceptor site, potentially leading to a transcript encoding a functionally defective protein.

To investigate whether mis-splicing occurred, RT-PCR was performed on cDNA from pools of WT, heterozygous, and homozygous mutant *nox2* zebrafish embryos. Although electrophoresis of RT-PCR products provided no evidence of intron retention (Fig. 1c), the products were sequenced to detect other forms of alternative splicing. This demonstrated that the mutation caused 20 bp of exon 3 sequence to be abnormally spliced out in the *nox2*^−/−^ zebrafish (Fig. 1, d and e). Although a mixture of normal and mis-spliced sequences was evident in heterozygous animals, only this mis-spliced transcript was detected by Sanger sequencing in homozygous mutant animals, suggesting that this is the dominant mis-spliced transcript. The mis-spliced sequence results in a frameshift leading to a premature stop codon, encoding a truncated Nox2 protein lacking the entire FAD and NADPH domains, with resultant loss of function. Hence, it was predicted that the homozygous mutants would be functionally Nox2-deficient.

### Impaired ROS production by *nox2* mutant myeloid cells

ROS production was first assessed by PMA-stimulated whole embryos. In this assay, ROS production in WT embryos was completely PMA-dependent, precluding interference from EGFP reporter genes carried in the genetic background (Supplementary Fig. 2a). PMA stimulated ROS activity in all WT embryos, with variation between individual embryos. (Supplementary Fig. 2a). In the context of this variation, in this whole embryo assay, both *nox2^+/−^* and *nox2^−/−^* embryos displayed a range of PMA-stimulated ROS production that overlapped with that of WT embryos (Supplementary Fig. 2b).

**Fig. 2.**
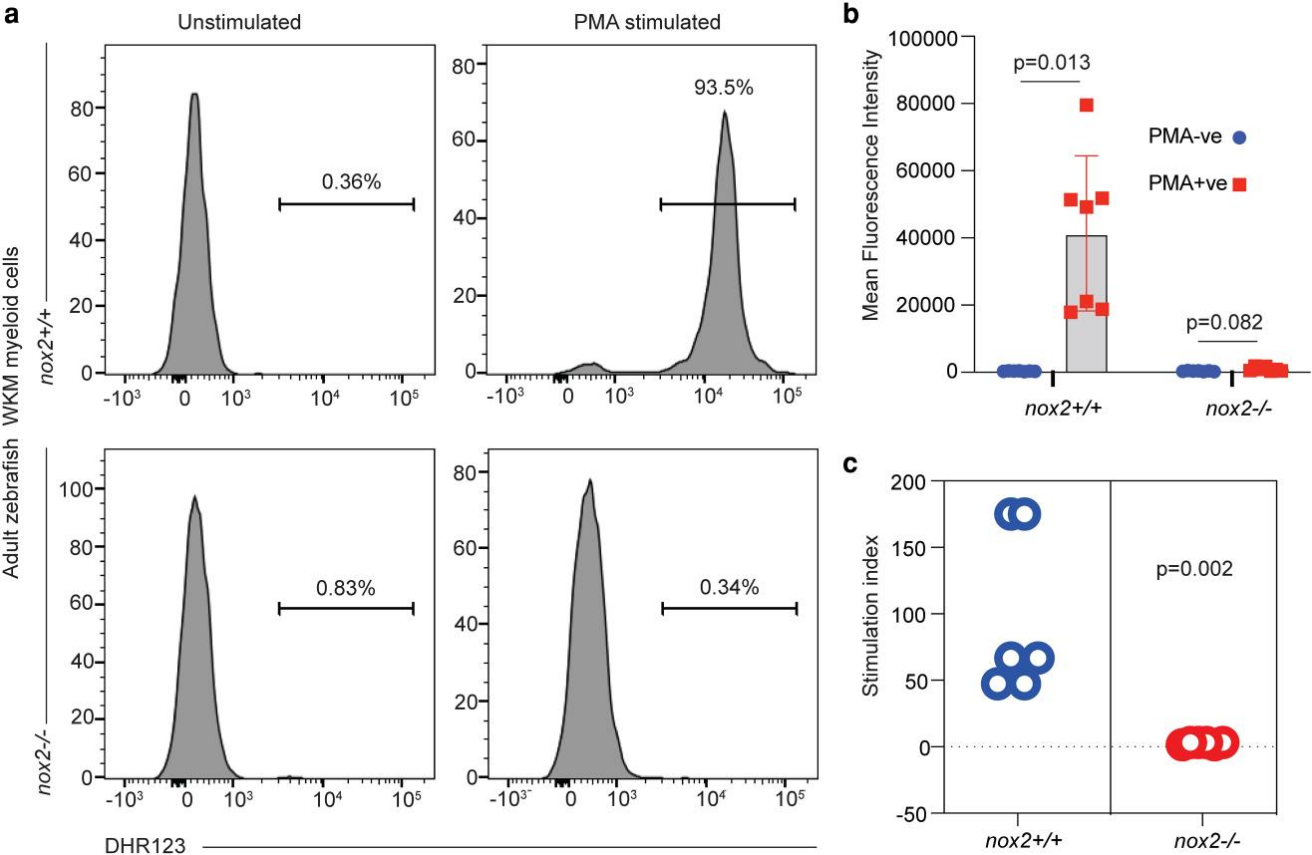
ROS deficiency in adult *nox2−/−* zebrafish myeloid cells. a) Representative histograms of DHR123 staining flow analysis in *nox2*+/+ (upper panels) and *nox2−*/− (lower panels) adult WKM myeloid cells. b) Comparison of MFI of DHR123 between unstimulated and PMA-stimulated myeloid cells from *nox2*+/+ and *nox2−*/− WKM. c) PMA stimulation index of ROS for *nox2*+/+ and *nox2−*/− WKM myeloid cells. DHR123, dihydrorhodamine 123; MFI, mean fluorescence intensity; WKM, whole kidney marrow; +/+, WT; −/−, homozygous mutant; *N*, *nox2+/+*, 7; *nox2−/−*, 7. Turkey's multiple comparison (b); Mann–Whitney (c); *P* < 0.05.

We hypothesized that the loss of Nox2-dependent ROS production in *nox2^−/−^* embryos would be predominantly in myeloid cells and would potentially be masked in whole embryo assays by widespread PMA stimulation of ROS production by other NADPH oxidases, including Nox1 (Kim *et al*. 2007), Nox3 (Ueno *et al*. 2005), and Nox5 (Jagnandan *et al*. 2007). ROS production was therefore assessed in myeloid cells specifically, using FACS-gated myeloid cells from adult WKM (gating strategy shown in Supplementary Fig. 3) and assessing ROS production by a flow cytometric assay based on the oxidative spectral shift of DHR123 following PMA stimulation of the myeloid cells oxidative burst. PMA-stimulated WT myeloid cells showed robust induction of ROS activity (Fig. 2, a and b). In contrast, PMA stimulation of *nox2^−/−^* myeloid cells induced no significant ROS activity (Fig. 2a–c). This demonstrates that the *nox2* mis-splicing mutation abrogates ROS activity in *nox2^−/−^* mutant myeloid cells including neutrophils.

### *Nox2* deficiency affects early embryo viability

**Fig. 3.**
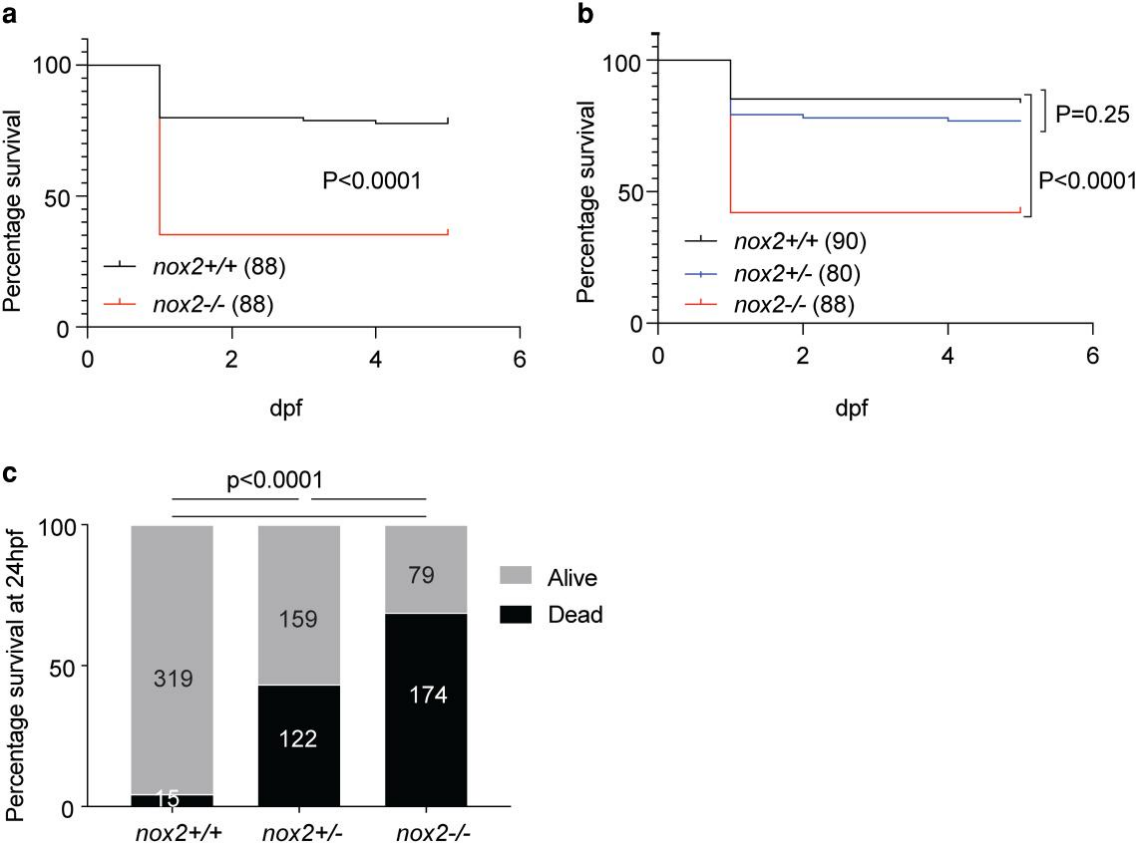
Reduced viability of embryos carrying the *nox2* mutant allele. a) Survival curve of concurrent cohorts of WT and *nox2−*/− zebrafish embryos. b) Survival curve of concurrent cohorts of WT, *nox2*+/−, and *nox2−*/− zebrafish embryos. c) Twenty-four (24) h viability of *nox2* WT, heterozygous, and homozygous zebrafish embryos. Panels a, b, and c were performed independently on different days and represent cohorts of genotype-identical embryos assayed in parallel. *N*-values in brackets (a, b) and within columns (c)—note that although *N*-values are similar, a and b are independent experiments; +/+, WT; +/−, heterozygous; −/−, homozygous. Log-rank (Mantel–Cox) tests (a, b), Fisher’s exact test (c), *P* < 0.0001.

Adult heterozygous and homozygous *nox2* mutants were recovered and displayed no gross phenotypic abnormalities. Both sexes were fertile as homozygous adults. However, while mating the *nox2* mutant, a lower-than-expected recovery of embryos carrying the *nox2* mutation for experiments suggested a high rate of early embryonic death. The effect of the *nox2* mutation on early embryo viability was therefore assessed in 5-day survival assays. Concurrent cohorts of WT, heterozygous, and homozygous *nox2* embryos were generated by appropriate crosses: WT to WT (WT × WT), WT to homozygous (WT × *nox2*^−/−^), and homozygous to homozygous (*nox2*^−/−^*×nox2^−/−^*) and monitored for viability. The survival of *nox2* homozygous mutants was significantly reduced, with all excess mortality occurring in the first 24 h post fertilization (hpf), regardless of parentage (Fig. 3a–c). The mechanism by which *nox2* directly affects early embryo survival is an interesting question for future studies.

### *Nox2* deficiency does not affect initial neutrophil recruitment to a wound

Despite their reduced early viability, enough *nox2* mutant embryos survive for testing biological questions. The arrival of neutrophils at sites of tissue injury is an initial step of acute inflammation, so we investigated the requirement for *nox2* in the initiation of acute inflammation in standard zebrafish injury assays. On the basis of previous reports of NOX2-driven “sterile” hyperinflammation in experimental mice and human patients, we hypothesized that sterile injury would induce excessive neutrophilic inflammation in the *nox2* zebrafish mutants (Dinauer 2019; Fernandez-Boyanapalli *et al*. 2010; Zeng *et al*. 2013).

**Fig. 4.**
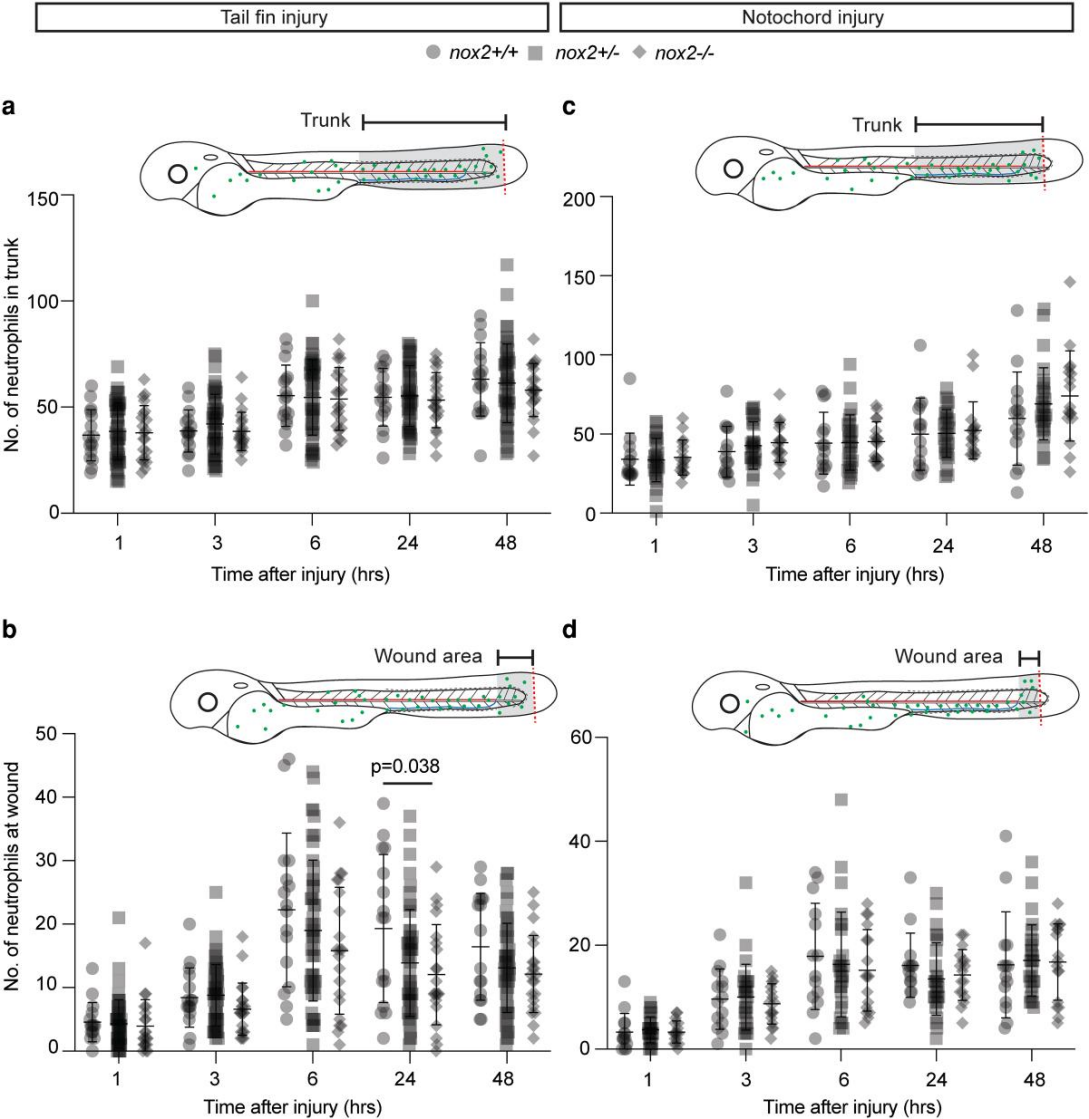
Acute neutrophil inflammatory response in *nox2* zebrafish embryos. a, c) Number of neutrophils throughout the trunk following tail fin injury (a) and notochord injury (c). b, d) Number of neutrophils at wound following tail fin injury (b) and notochord injury (d). Tail fin injury, *N* = 16(+/+), 50(+/−), and 21(−/−); notochord injury, *N* = 14 (+/+), 28 (+/−), and 17 (−/−); WT; +/−, heterozygous; −/−, homozygous. Pooled data of 3 independent experiments. Bars indicate mean ± SD. Two-way ANOVA with Tukey's multiple comparisons test, *P* < 0.05.

The temporal profile of neutrophil recruitment to a simple tail fin transection injury is a well-established assay of neutrophil chemotaxis (Renshaw *et al*. 2006). The *nox2* mutation is carried on transgenic reporter backgrounds allowing neutrophil number and distribution to be easily quantified. The *nox2*^−/−^ embryos had normal and unchanging numbers of trunk neutrophils available for initial relocation to a wound and throughout the 3 h assay (Fig. 4a). Compared with WT, there was no consistent statistically significant difference in wound zone neutrophil numbers in *nox2*^+/−^ and *nox2^−^*^/−^ embryos, although a transient statistically significant decrease of uncertain biological significance was observed at 24 h postwounding (hpw) (Fig. 4b).

The magnitude of neutrophil migration to tailfin wounds is dependent on the degree of injury, with greater neutrophil inflammatory responses following injuries involving the notochord as well as the fin (Miskolci *et al*. 2019). We therefore also assessed initial neutrophil recruitment following a larger and more severe tail fin injury involving the tip of the notochord, but again, there was no statistically significant difference between WT and *nox2* mutants (Fig. 4, c and d).

These 3 h assays provided no evidence of augmented initial neutrophil recruitment to an acute sterile injury in *nox2* deficiency states.

## Discussion

We have generated a new *nox2* loss-of-function zebrafish mutant by gene editing resulting in a mis-spliced *nox2* transcript. Although it remains theoretically possible that this allele, being a splicing mutant, is a hypomorph rather than an absolute null, it is a severely compromising allele and appears to be a functional null. No WT transcript signal was discernable in Sanger sequencing chromatograms of cDNA pools prepared from pools of *nox2^−/−^* mutant embryos (Fig. 1d), suggesting that this single mis-splicing event was predominant. RNA-seq would be a more sensitive way of detecting other minor mis-spliced transcripts. The *nox2^−/−^* mutant adult myeloid cells demonstrated markedly reduced ROS production with a near-zero stimulation index in a standard clinical assay used for CGD diagnosis, functionally validating this allele as a model for the most common form of CGD. *NOX2* mutants account for ∼65% of CGD in human patients (Dinauer 2019).

There are many in vitro studies of NOX2 mutant or NOX-2 functionally compromised mammalian cells but only a few animal models (Dinauer 2019). A murine model replicated the X-linked inheritance of human *NOX2* CGD and displayed the classical CGD disease features of infection vulnerability and an enhanced acute neutrophil response to “sterile” inflammation (Pollock *et al*. 1995). Two zebrafish *nox2* mutant alleles were reported in a study investigating a *nox2* requirement in retino-tectal development (Weaver *et al*. 2018). However, despite the truncating frameshift mutations predicting a severely functionally compromised protein, neither an in vivo biosensor approach nor an in vitro analysis of cultured retinal ganglion cells was able to detect a significant alteration in hydrogen peroxide dynamics in these mutants in this initial study, although a subsequent study demonstrated a defect (Terzi and Suter 2020).

However, these 2 previously reported zebrafish *nox2* mutant alleles have not been characterized for their effects on myeloid cell ROS production. The functional validation of their ROS production impairment is based on altered H_2_O_2_ dynamics in ex vivo dissociated culture of retinal ganglion cells (Terzi *et al*. 2021; Weaver *et al*. 2018). A *p22^phox^/cyba* zebrafish mutant has been studied for its fungal infection susceptibility; although no direct experimental demonstration of impaired neutrophil ROS production was provided, a neutrophil-specific *p22^phox^/cyba* rescue reversed a phenotype attributed to ROS deficiency (Schoen *et al*. 2019). Morphants have also been used to study transient loss of phagocyte NOX subunits, including *nox2/cybb* (Bernut *et al*. 2019; Brothers *et al*. 2011; Mesureur *et al*. 2017; Razaghi *et al*. 2018; Roca and Ramakrishnan 2013; Yang *et al*. 2012), *p47^phox^/ncf1* (Brothers *et al*. 2013; Brothers *et al*. 2011; Mesureur *et al*. 2017; Phan *et al*. 2018; Sipka *et al*. 2021; Yang *et al*. 2012), and *p22^phox^/cyba* (Prajsnar *et al*. 2021; Schoen *et al*. 2019).

Our initial phenotypic characterization does not yet permit direct comparison between adult and larval ROS requirements in the *nox2* mutant. We have shown that this new *nox2* mutant severely impairs ROS production by adult myeloid cells. However, this has not yet been shown experimentally for embryonic myeloid cells. This could be done by ROS assays on FACS-gated myeloid cell populations from embryos or in vivo by using cell-specific ROS reporters such as *lyz*:Hyper (Pase *et al*. 2012). The impaired survival of *nox2*-deficient larvae has been documented up to 5 dpf, but whether there is further attrition of *nox2^−/−^* animals into adult life has not been examined.

The multimeric phagocyte NADPH oxidase enzymatic complex is also of interest as the prototypic NADPH oxidase, and its NOX2 gp91^phox^/CYBB subunit has provided a paradigm for understanding the structure and function of other NADPH oxidase enzyme complexes (NOX 1, 3–5, and also DUOX 1-2) (Quinn 2013). Our attempts to validate functional impairment of ROS generation highlight the need to consider both the specificity of the stimulus and the cell types expressing the NADPH oxidase subunit of interest. Although NOX2 is sometimes regarded as a neutrophil-specific PHOX subunit, no individual NADPH oxidase subunit is truly exclusively specific to 1 cell type (Quinn 2013). Similarly, while PMA is a potent stimulator of neutrophils (Degroote *et al*. 2019; Karlsson *et al*. 2000), in the context of the whole animal, it should not be considered to be exclusively a stimulator of ROS produced by the phagocytic oxidative burst (Quinn 2013).

Hyperinflammation characterized by excessive neutrophil infiltrates and granulomata is a hallmark of CGD (Dinauer 2019) and is considered to represent a dysfunctional neutrophil inflammatory response in the absence of ROS. However, the mechanisms underpinning this phenomenon are not fully understood. Zebrafish are an ideal model for studying inflammation, as the entire process can readily be visualized in vivo (Robertson and Huttenlocher 2022), and the acute neutrophil response to stereotypic injuries is well described (Miskolci *et al*. 2019; Pase *et al*. 2012; Renshaw *et al*. 2006). A cell-intrinsic neutrophil requirement for mitochondrial-generated ROS for normal velocity neutrophil migration has been demonstrated (Zhou *et al*. 2018). Schoen *et al.* (2019) demonstrated an abnormally sustained neutrophil migration response to a fungal infection challenge in a *p22^phox^/cyba* mutant, using a neutrophil-specific rescue strategy to demonstrate a contribution of a cell-intrinsic neutrophil ROS. Both observations support the hypothesis that there is a cell-autonomous requirement for ROS regulating neutrophil migration. However, we did not observe a quantitative perturbation in the accumulation of neutrophils to 2 types of “sterile” injury in our *nox2* mutant in the first 3 days after injury, despite the statistical power of pooled group sizes of *n* > 80. It may be that the migratory response needs to be examined over a longer timeframe or that migration to an ostensibly “sterile” surgical wound involves different mechanisms to those of neutrophil wandering or migration to a fungal infection focus or zymosan-induced “sterile” inflammation (Fernandez-Boyanapalli *et al*. 2010; Schoen *et al*. 2019). It may also reflect that the cumulative outcome of the different extents and degrees of ROS production impairment differs in each of these models which are directed at different NADPH oxidase subunits and use different techniques, due to variation in either the full distribution of cell types that have impaired ROS production or in the potential leakiness of the different genetic methodologies. It is also theoretically possible that maternal rescue of the heterozygous and homozygous *nox2^−/−^* migration phenotype occurred, as the embryos resulted from an incross of heterozygotes, although we are unaware of any evidence that there is maternal deposition of *nox2* transcripts or protein. Furthermore, our expression analysis shows that *nox2* expression is exclusively strong in a myeloid population (marked by neutrophil and macrophage markers) amongst all unselected cells present in the injured and uninjured caudal fin of zebrafish larvae (Supplementary Fig. 1b). Experiments to explore these interesting possibilities fell beyond the scope of this initial study.

This new *nox2* mutant provides a valuable tool for further exploring this and other *nox2*-dependent phenotypes causing morbidity and mortality for CGD patients.

## Supplementary Material

jkae079_Supplementary_Data

## Data Availability

Strains are available upon request. A reagent table is provided as supplementary data. The authors affirm that all data necessary for confirming the conclusions of the article are present within the article, figures, and supplementary figures. Supplemental material available at G3 online.
