# Supplementary material for: A nox2/cybb zebrafish mutant with defective myeloid cell reactive oxygen species production displays normal initial neutrophil recruitment to sterile tail injuries
Source: G3 (Bethesda). 2024 May 2;14(6):jkae079. doi: 10.1093/g3journal/jkae079 (PMC11152067; doi:10.1093/g3journal/jkae079)
Supplement: jkae079_Supplementary_Data [file jkae079_supplementary_data.pdf]

Figure S1

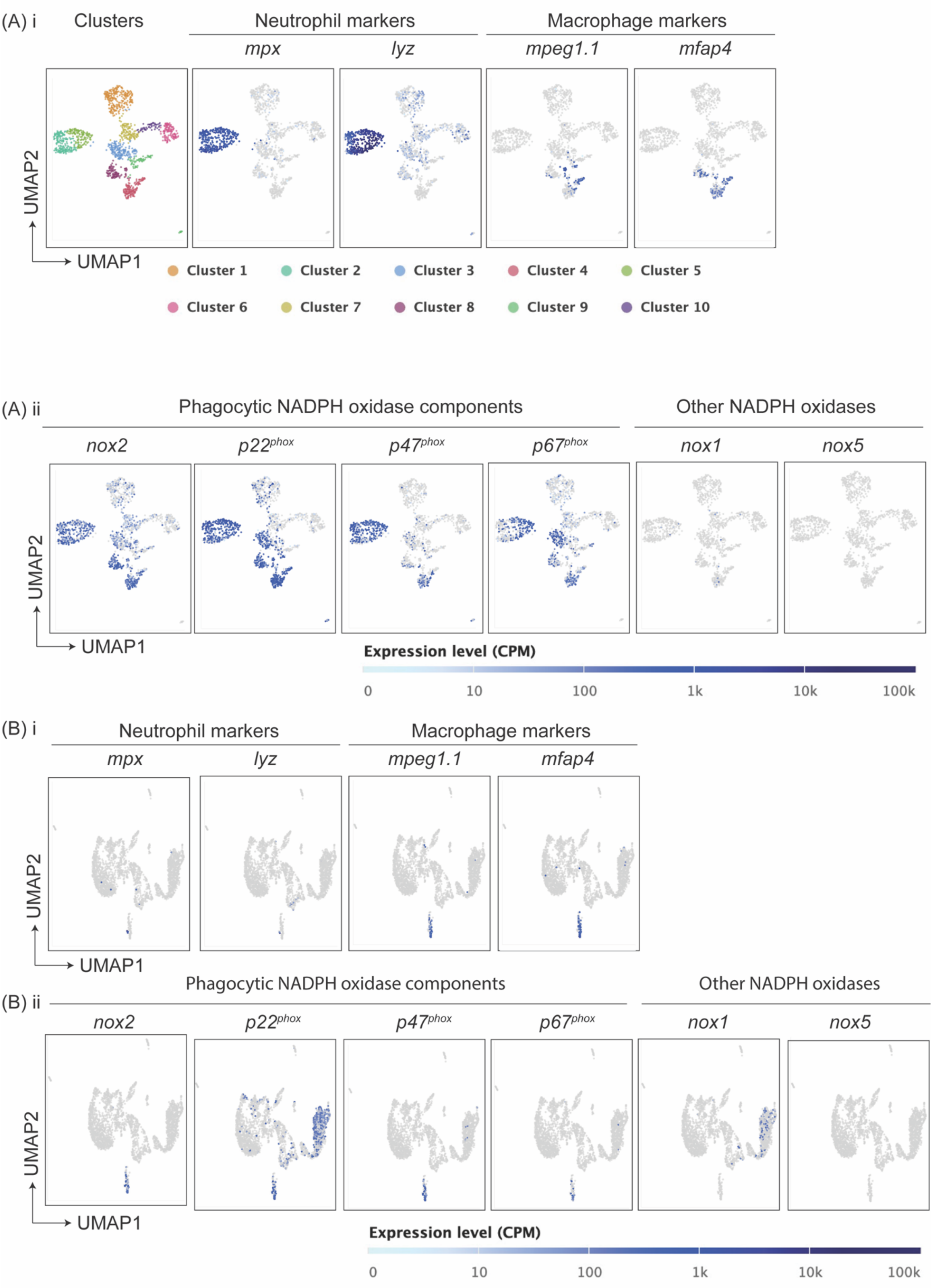
(A) i
Clusters
Neutrophil markers
mpx
lyz
Macrophage markers
mpeg1.1
mfap4
UMAP2
UMAP1
Cluster 1
Cluster 2
Cluster 3
Cluster 4
Cluster 5
Cluster 6
Cluster 7
Cluster 8
Cluster 9
Cluster 10
(A) ii
Phagocytic NADPH oxidase components
nox2
p22phox
p47phox
p67phox
Other NADPH oxidases
nox1
nox5
UMAP2
UMAP1
Expression level (CPM)
0
10
100
1k
10k
100k
(B) i
Neutrophil markers
mpx
lyz
Macrophage markers
mpeg1.1
mfap4
UMAP2
UMAP1
(B) ii
Phagocytic NADPH oxidase components
nox2
p22phox
p47phox
p67phox
Other NADPH oxidases
nox1
nox5
UMAP2
UMAP1
Expression level (CPM)
0
10
100
1k
10k
100k

Figure S1. Expression of zebrafish *nox2* and other NADPH oxidase components with reference to myeloid cell populations

A. Adult kidney single cell expression data.
   (i) Cells of individual phagocyte cell lineages identified by expression of zebrafish neutrophil-lineage markers (*mpx*, *lyz*) and macrophage-lineage markers (*mpeg1.1*, *mfap4*).
   (ii) Expression of zebrafish *nox2/cybb*, *ncf1/p47*^{phox}, *ncf2/p67*^{phox}, *p22*^{phox}*/cyba*, *nox1* and *nox5* in the same population of single cells. Among blood cells, *nox2* is expressed, *nox1* or *nox5* are not expressed, and the cell populations expressing the four NADPH oxidase subunits overlap.

B. Tail injury single cell expression data.
   (i) Cells of individual phagocyte cell lineages identified by expression of zebrafish neutrophil-lineage markers (*mpx*, *lyz*) and macrophage-lineage markers (*mpeg1.1*, *mfap4*).
   (ii) In this heterogeneous population of cells, *nox2* expression is confined to those cells marking as phagocytes in (i), whereas *nox1* is expressed in another cell type. The expression of *ncf1/p47*^{phox}, *ncf2/p67*^{phox} is largely coincident with *nox2*, but *p22*^{phox}*/cyba* is expressed in a more diverse population of cells and cannot be regarded as a myeloid-restricted NADPH oxidase component.

UMAP, Uniform Manifold Approximation and Projection; Single cell analysis data from EMBL-EBI Elixir node Single Cell Expression Atlas (for details, see Materials and Methods).

Figure S2

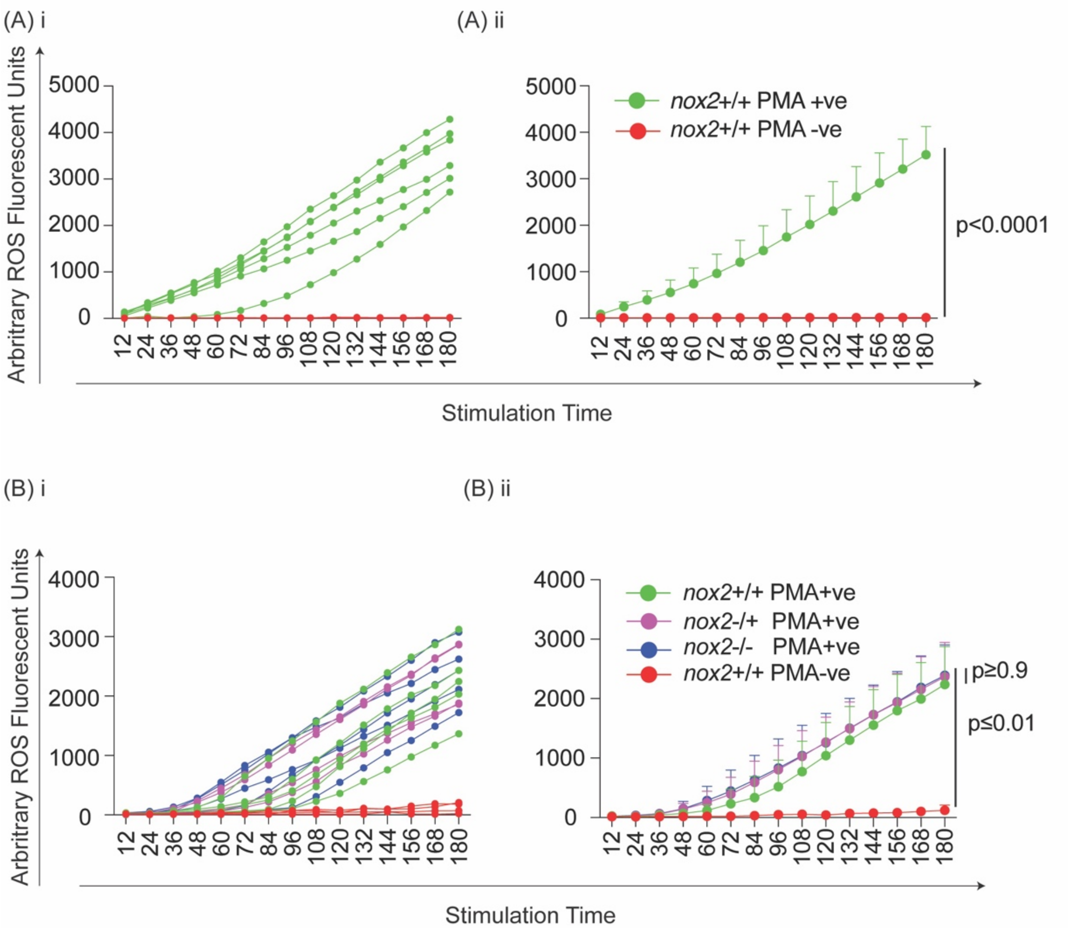

Figure S2. Heterogeneity of ROS production in whole zebrafish embryos

A. ROS levels in individual wild type zebrafish embryos (i) and mean ROS production (ii), with and without PMA stimulation. *nox2*$^{+/+}$ PMA-ve, n=6; *nox2*$^{+/+}$ PMA+ve, n=6.

B. ROS levels of individual *nox2*$^{+/+}$, *nox2*$^{+/-}$ and *nox2*$^{-/-}$ zebrafish embryos (i) and mean ROS production (ii) with and without PMA stimulation. *nox2*$^{+/+}$ PMA-ve, n=4, *nox2*$^{+/+}$ PMA+ve, n= 5, *nox2*$^{+/-}$ PMA+ve, n=4; *nox2*$^{-/-}$ PMA+ve, n=5.

Figure S3

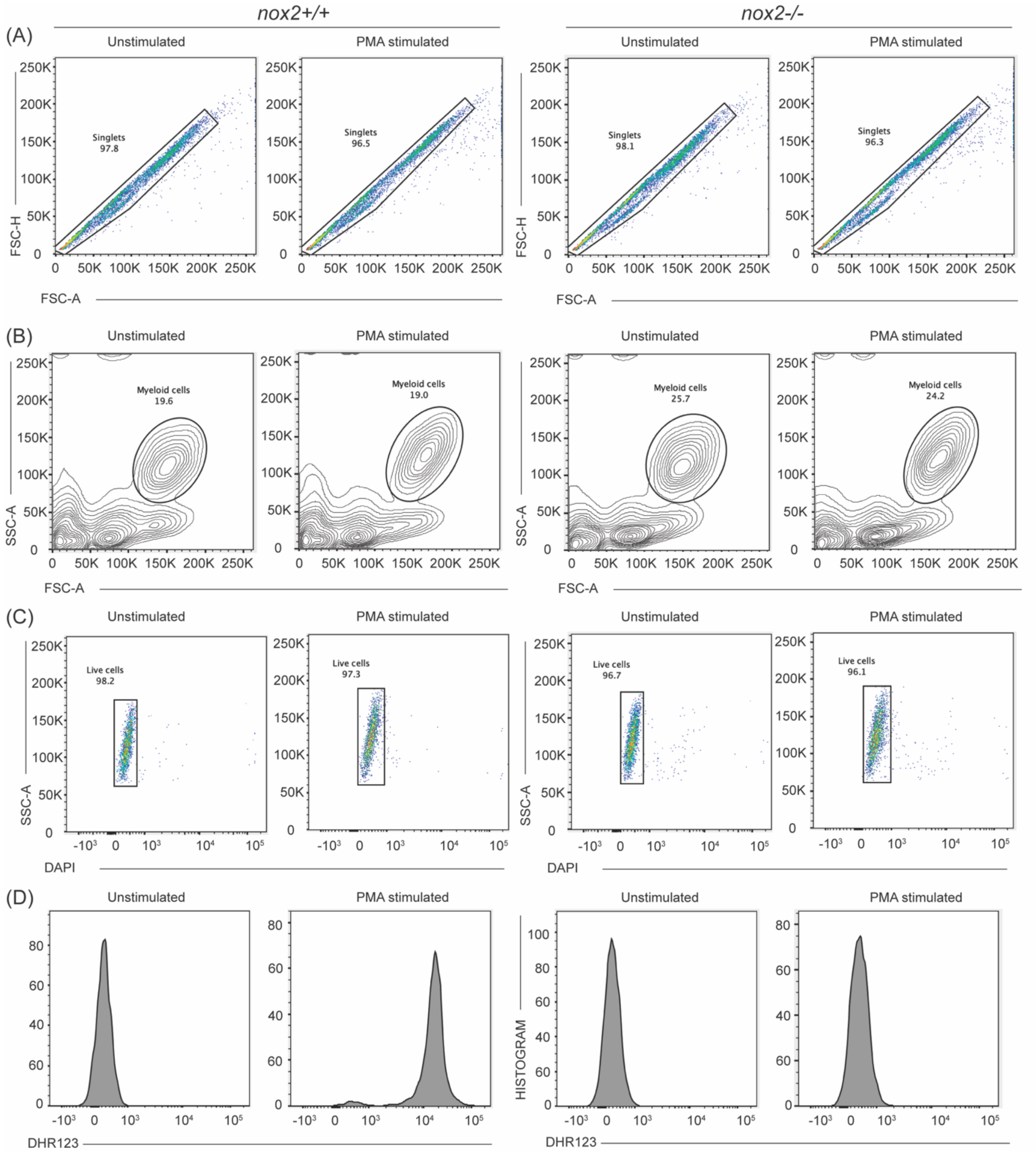

Figure S3. Flow analysis gating strategies for myeloid cell populations (supports Figure 2).

Representative wild type *nox2+/+* and mutant *nox2-/-* zebrafish whole kidney marrow samples, with and without PMA stimulation, showing gating for: (A) single cells; (B) FSC^{hi} SSC^{hi} (encircled) myeloid cells; (C) DAPI negative live cells; (D) DHR123 profile. Abbreviations: DHR123, dihydro rhodamine 123; WKM, whole kidney marrow
